# Identification of SARS-CoV-2 Main Protease (Mpro) Cleavage Sites Using Two-Dimensional Electrophoresis and In Silico Cleavage Site Prediction

**DOI:** 10.3390/ijms24043236

**Published:** 2023-02-06

**Authors:** Noémi Miltner, Gergő Kalló, Éva Csősz, Márió Miczi, Tibor Nagy, Mohamed Mahdi, János András Mótyán, József Tőzsér

**Affiliations:** 1 Department of Biochemistry and Molecular Biology, Faculty of Medicine, University of Debrecen, 4032 Debrecen, Hungary; 2 Proteomics Core Facility, Department of Biochemistry and Molecular Biology, Faculty of Medicine, University of Debrecen, 4032 Debrecen, Hungary; 3 Department of Applied Chemistry, Faculty of Science and Technology, University of Debrecen, 4032 Debrecen, Hungary

**Keywords:** coronavirus, SARS-CoV-2, main protease, Mpro, NetCorona, cleavage site identification, cleavage site prediction, host protein cleavage, specificity, two-dimensional gel electrophoresis

## Abstract

The main protease (Mpro) of severe acute respiratory syndrome coronavirus 2 (SARS-CoV-2) plays a crucial role in its life cycle. The Mpro-mediated limited proteolysis of the viral polyproteins is necessary for the replication of the virus, and cleavage of the host proteins of the infected cells may also contribute to viral pathogenesis, such as evading the immune responses or triggering cell toxicity. Therefore, the identification of host substrates of the viral protease is of special interest. To identify cleavage sites in cellular substrates of SARS-CoV-2 Mpro, we determined changes in the HEK293T cellular proteome upon expression of the Mpro using two-dimensional gel electrophoresis. The candidate cellular substrates of Mpro were identified by mass spectrometry, and then potential cleavage sites were predicted in silico using NetCorona 1.0 and 3CLP web servers. The existence of the predicted cleavage sites was investigated by in vitro cleavage reactions using recombinant protein substrates containing the candidate target sequences, followed by the determination of cleavage positions using mass spectrometry. Unknown and previously described SARS-CoV-2 Mpro cleavage sites and cellular substrates were also identified. Identification of target sequences is important to understand the specificity of the enzyme, as well as aiding the improvement and development of computational methods for cleavage site prediction.

## 1. Introduction

The severe acute respiratory syndrome coronavirus 2 (SARS-CoV-2) is the causative agent of coronavirus disease-19 (COVID-19). Since its identification in December 2019 in Wuhan (China) [1], the pandemic has reportedly been associated with over 753.8 million confirmed cases of infections and more than 6.8 million infection-related deaths worldwide, according to the World Health Organization (WHO).

The SARS-CoV-2 genome encodes two cysteine proteases, a papain-like (PLpro) and a 3-chymotrypsin-like (3CL) protease, and the latter one is also referred to as the main protease (Mpro) [2]. The viral proteases are responsible for processing the polyproteins into functional units via limited proteolysis [3,4]. Due to their essential contribution to the viral life cycle, viral proteases became one of the important targets of antiviral therapies. Paxlovid, an oral antiviral drug composed of a combination of nirmatrelvir (a peptidomimetic SARS-CoV-2 Mpro inhibitor) and the booster ritonavir (HIV-1 protease inhibitor that is able to inhibit Cyp450), is the first potent SARS-CoV-2 Mpro-specific antiviral that has been authorized for the treatment of non-hospitalized COVID-19 adult patients [5].

The viral proteases are able to cleave not only viral but also host proteins. Processing the infected cell’s proteins may aid multiple viruses in completing their replication cycle and evading immune responses, contributing to their pathogenesis. This has already been described for multiple viral proteases such as HIV-1 [6], alphaviruses [7,8], and coronaviruses [9,10].

Identifying host substrates is required to better understand viral pathogenesis, and experimental and in silico approaches are indeed available for this purpose. Different sequence-based in silico approaches can be applied for the prediction of cleavage sites of SARS-CoV-2 protease. For example, the potential target sites can be estimated by analysis of the short stretches of homologous host-pathogen protein sequences (SSHHPS) [7,11]. This method—which is based on the full or partial sequence identity shared by the viral and host proteins—has already been applied to predict host targets of the SARS-CoV-2 enzymes, such as the PLpro [9] and the Mpro [12]. Another approach is used by the NetCorona 1.0 web server: the potential cleavage sites are predicted based on analysis of consensus cleavage sites which were established based on the known cleavage site sequences, using a neural network model [13]. The NetCorona online tool has been primarily designed for the prediction of SARS-CoV Mpro cleavage sites. The recently developed 3CLP online tool (“an online tool for predicting coronavirus 3CL protease cleavage sites”) using a random forest-based method can also be applied for the estimation of Mpro cleavage sites, but this tool is not specific for any coronavirus enzyme. It can be used to predict the target sequences of multiple coronavirus main proteases based on the identification of the highly conserved cleavage site motif [14].

Currently, multiple experimental data are available for those cellular proteins that are also substrates of SARS-CoV-2 Mpro in infected cells. Some studies revealed the processing of proteins that are involved in the regulation of inflammation, such as NACHT, LRR, and PYD domains-containing protein 12 (NLRP12) [15], Ring finger protein 20 (RNF20) [16], and selective autophagy receptor p62 [17] were also identified as host substrates of Mpro. In addition, large-scale proteomic approaches, such as N-terminomics, also enabled the identification of multiple SARS-CoV-2 Mpro substrates, which are connected to various pathways and processes [18,19,20]. Identification of new substrates and cleavage sites is important to determine the specificity of SARS-CoV-2 Mpro and understand the viral pathogenesis. More efficient predictors may be developed based on the experimentally determined cleavage site sequences.

In this paper, we describe the effect of SARS-CoV-2 Mpro expression on the proteome of HEK293T cells using two-dimensional difference gel electrophoresis (2D-DIGE). 2D-DIGE is a modified form of two-dimensional electrophoresis (2DE) in which two or more samples—labeled with different cyanine dyes—can be analyzed on the same gel [21]. The sequences of candidate cellular substrates of Mpro were analyzed in silico, followed by a comparison of the cleavage probabilities predicted by the NetCorona 1.0 and 3CLP web servers. Cleavage site sequences that were identified by NetCorona were then inserted into His_6_-MBP-mEYFP recombinant protein substrates, followed by cleavage reactions and determination of cleavage positions. Our work may help to better understand the specificity of SARS-CoV-2 Mpro and its potential involvement in pathogenesis via the identification of cleavage site sequences and cellular host substrates.

## 2. Results

### 2.1. Cell Culturing and Transfection

**Figure 1:**
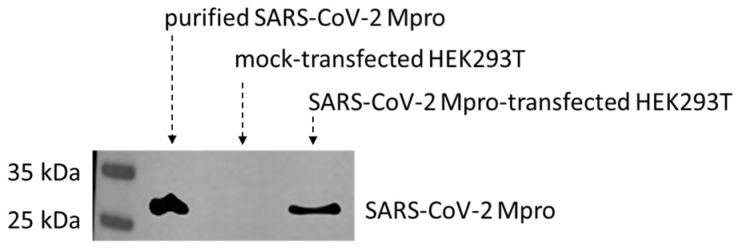
Expression of SARS-CoV-2 Mpro in transfected HEK293T cells. SARS-CoV-2 Mpro was detected in the lysates of transfected HEK293T cells. The recombinant protein was absent from the mock-transfected cells. Purified His_6_-SARS-CoV-2 Mpro was used as a positive control.

To identify cellular substrates of the SARS-CoV-2 Mpro, we studied human HEK293T cells expressing the recombinant enzyme. First of all, expression of SARS-CoV-2 Mpro in the transfected cells was confirmed by Western blot using an anti-Mpro antibody. The expression of Mpro was successfully detected 24 h post-transfection. The enzyme was absent from the lysate of the mock-transfected cells (Figure 1). Purified SARS-CoV-2 Mpro was used as a positive control [12].

### 2.2. Two-Dimensional Difference Gel Electrophoresis and Protein Identification

**Figure 2:**
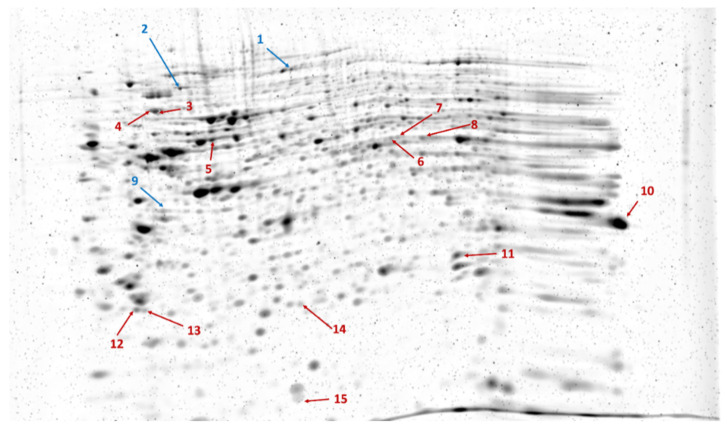
Representative image of RuBPS-stained gel showing the spots separated by 2DE. The image was generated by the Delta2D 4.8.2 software by warping gel images. Arrows indicate spots with significantly decreased (blue) or increased (red) intensity in SARS-CoV-2 Mpro-transfected cells compared to those of mock-transfected cells, based on the analysis of three parallel gels.

The proteomes of the transfected cells were subjected to 2D-DIGE analysis. After separation, the gels were scanned, followed by image analysis. After warping, the fused image containing spots from all Cy-dye labeled gels was generated. In total, 339 spots were detected. Statistical analysis revealed 15 spots showing statistically significant differences between SARS-CoV-2 Mpro and mock-transfected samples. As compared to the control, only three spots showed decreased intensity (ratio: 0.49–0.87), the other spots had increased intensity (ratio: 1.43–3.96) in the SARS-CoV-2 Mpro-transfected samples (Figure 2). Proteins identified in the spots and excised from the gel are listed in Table 1. 

### 2.3. SARS-CoV-2 Mpro Cleavage Site Prediction

All of the proteins identified in such spots that showed statistically significant differences were subjected to sequence-based cleavage site prediction. The prediction was performed using NetCorona 1.0 [13] and 3CLP web servers [14], which have been designed for the prediction of coronavirus Mpro cleavage sites. 

The NetCorona and 3CLP algorithms identified a different number of potential cleavage sites, and the cleavage probabilities predicted for the same cleavage sites also showed considerable differences in some cases (Table 1). Based on NetCorona prediction, 13 target sequences were considered to be candidate SARS-CoV-2 Mpro cleavage sites, 6 of these sites were identified only by NetCorona, while 7 sites were predicted by the 3CLP tool as well. NetCorona scores obtained for the selected target sites ranged from 0.51 to 0.92, indicating various cleavage probabilities (Table 1). 

Neutral alpha-glucosidase AB (GANAB), stathmin (STMN1), ELAV-like protein 1 (ELAV1), SUMO-activating enzyme subunit 2 (SAE2), and the ubiquitin-like modifier-activating enzyme 1 (UBA1) proteins have already been identified as substrates of SARS-CoV-2 Mpro in vitro [19] (Table 2). Both the NetCorona and 3CLP algorithms failed to identify SARS-CoV-2 Mpro cleavage sites that were identified previously in GANAB (Q343*G344) and SAE2 (Q82*F83) proteins [19] and a known cleavage site of STMN1 protein was not predicted by the 3CLP method (Table 1). Interestingly, none of the herein-identified candidate targets were previously identified by SSHHPS analysis, which was performed based on the comparison of sequences of human proteins and SARS-CoV-2 autoproteolytic cleavage sites [12]. No such sites were identified experimentally by Koudelka et al. [19]. However, they were predicted in this work by the 3CLP algorithm. 

### 2.4. Cleavage Reactions and Cleavage Site Identification

**Figure 3:**
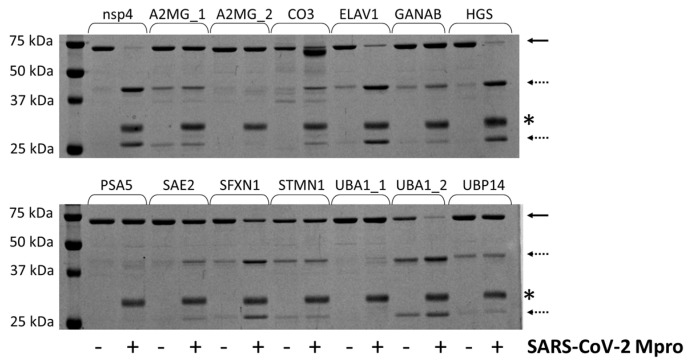
Cleavage of recombinant substrates by SARS-CoV-2 Mpro. The representative gel image shows SDS-PAGE analysis of the recombinant His_6_-MBP-mEYFP substrates after incubation with or without SARS-CoV-2 Mpro. The continuous and dashed arrows indicate the full-length substrates and the cleavage products, respectively. An asterisk indicates the band of the enzyme. The nsp4 indicates the substrate representing the TSAVLQ*SGFRKM autoproteolytic cleavage site of SARS-CoV-2 polyprotein [12].

In order to determine whether the candidate sites are processed by SARS-CoV-2 Mpro, we designed His_6_-MBP-mEYFP substrates. The recombinant proteins represented such sequences which were identified by the NetCorona algorithm as potential cleavage sites. Only those sequences were considered potential cleavage sites for which the sequence-based prediction resulted in >0.5 NetCorona score, sites that were not predicted to be cleaved by the SARS-CoV-2 Mpro (having <0.5 NetCorona score) were omitted from the in vitro analyses. We expected a correlation between the predicted scores and the in vitro cleavage efficiencies. The recombinant substrates were designed, prepared, and purified based on the protocols described previously [12,29]. The fusion proteins represented the P5-P5’ residues of the putative cleavage site sequences, and each contained Gln in the P1 position. A His_6_-MBP-mEYFP protein containing a natural autoproteolytic cleavage site of Mpro was used as a positive control substrate (Figure 3). 

The positive control substrate was the most efficiently cleaved. This recombinant protein was completely processed by SARS-CoV-2 Mpro. Only the turnovers of the ELAV1, HGS, and UBA1_2 substrates were highly comparable to that of the nsp4 substrate, while most of the investigated cleavage sites were cleaved with remarkably lower efficiency (Figure 3). Previously, we similarly observed low turnover for the human C-terminal-binding protein 1 (CTBP1) using the same experimental approach. Nevertheless, we successfully detected the processing and determined the cleavage position by MS-based analysis despite the low cleavage efficiency [12]. The His_6_-MBP-mEYFP substrates representing UBP14, UBA1_1, or PSA5 cleavage sites were not processed, and we observed no detectable product formation. Interestingly, the NetCorona web server predicted these sites as potential cleavage sites (with the lowest scores of the putative sites), while the 3CLP did not identify them as potential sites (Table 1).

**Figure 4:**
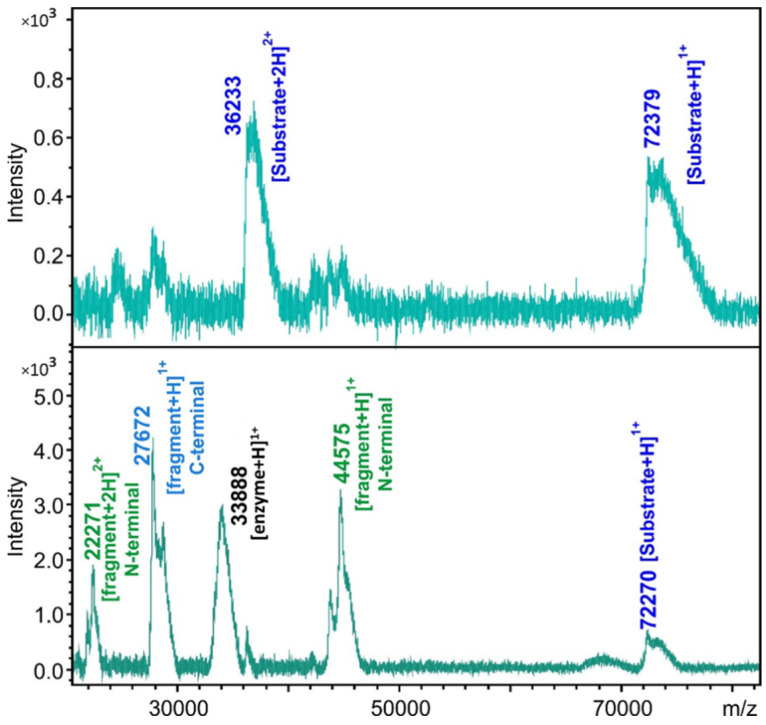
Cleavage site determination by MALDI-TOF MS. The representative spectra show the analysis of the substrate control (**upper panel**) and cleavage reaction (**lower panel**) in the case of His_6_-MBP-mEYFP substrate containing the SAE2 cleavage site.

The substrate controls and the cleavage reactions were also subjected to MALDI-TOF MS analysis (Figure 4). The molecular weights of the substrates and cleavage products were also determined, and the cleavage positions were identified based on the calculated and measured molecular masses (Table S1). The calculated and experimentally determined molecular masses were in good agreement, and the existence of the predicted cleavage positions was proved for those cleavage sites which were processed in vitro. Product formation was not detectable in the case of UBP14, UBA1_1, and PSA5 cleavage sites, even after using MALDI-TOF MS analysis of the cleavage reactions.

### 2.5. Structural Coordinates

**Figure 5:**
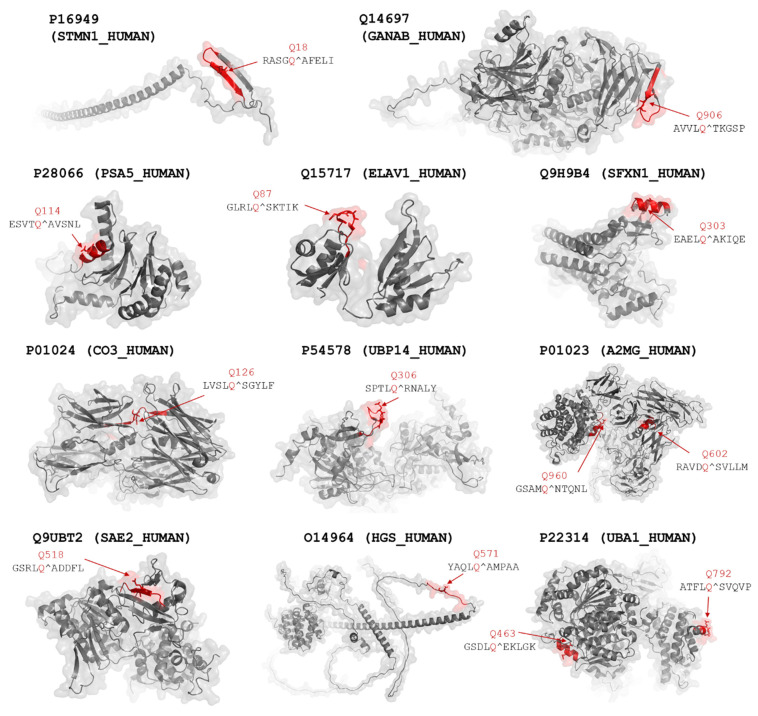
Structures of potential proteolytic targets of SARS-CoV-2 Mpro. Cleavage sites that were identified using NetCorona 1.0 web server are highlighted in red. P1 Gln residues of the cleavage sites are represented by sticks and shown by the arrows. Predicted cleavage probabilities are shown in Table 1.

The cleavage sites are structurally accessible in the His_6_-MBP-mEYFP substrates [30], but these sites may not necessarily be available for proteolysis in the properly folded full-length proteins. Therefore, we investigated the protein structures and the apparent accessibilities of the putative cleavage sites. Although the surface accessibilities of the residues were not quantified, the structural coordinates can be used to estimate whether the candidate sites are buried or located in the protein’s core. We found that most of the predicted sites are exposed on the protein surfaces (Figure 5). 

**Figure 6:**
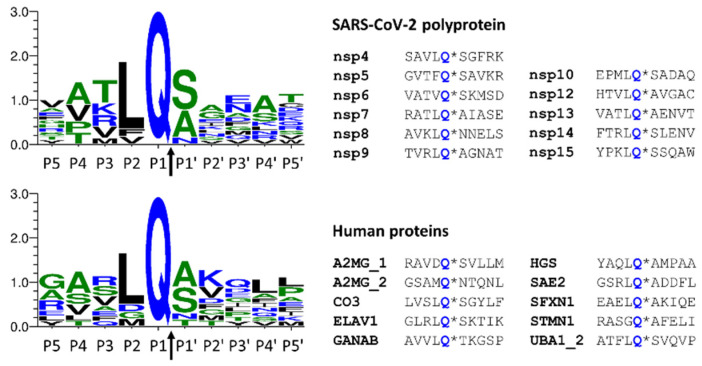
Comparison of cleavage site sequences. Autoproteolytic cleavage sites of SARS-CoV-2 polyprotein are shown, and the cleavage sites of Mpro identified in this work are also represented. The cleavage site sequences (P5-P5′ residues) of the viral polyprotein are shown based on Miczi et al. [12], and the sequences of human proteins are based on this study. The sequence logos were prepared using Weblogo 3.7.11 web server [31]. The arrows indicate the cleavage position between the P1 and P1′ residues.

The UBA1_2 site has already been identified as a target site of SARS-CoV-2 Mpro (Table 1). Thus, the native protein may be cleaved at this position, possibly due to the higher surface accessibility of this cleavage site. In contrast to this, we observed no cleavage at the UBA1_1 site of the recombinant multidomain substrate, which implies that the UBA1 protein is not processed at this site. The lowest NetCorona scores were predicted for the UBA1_1, UBP14, and PSA5 sites (0.506–0.541), indicating that the values being very close to the threshold (0.5) may be much less preferred recognition sites, and these sites are not expected to be processed efficiently in the natively folded proteins in vivo by SARS-CoV-2 Mpro. The recombinant substrates encompassing the predicted A2MG cleavage sites were processed in vitro by SARS-CoV-2 Mpro (Table 1). Although both candidate cleavage sites appear to have limited accessibility in the crystal structure of the A2MG protein (Figure 6), these motifs may potentially become accessible via domain movements and conformational changes of the full-length protein. Similar to most proteases, SARS-CoV-2 Mpro may also cleave peptide bonds with remarkably lower probability within α-helices. However, the in vitro processing of sites that were predicted to be located in helical regions (e.g., SFXN1) implies that the secondary structural arrangements of the target sites may be different in solution than in the crystal structures or structural models, and the conformational changes may potentially make the sites accessible for proteolytic processing. 

Sequences that were found to be processed by SARS-CoV-2 Mpro were compared to those of the autoproteolytic cleavage sites of the viral polyprotein (Figure 6). As was expected, the sequence logos were found to be highly similar because the applied in silico approaches are based on the analysis of consensus cleavage site sequence motifs. All the predicted sequences contain Gln in the P1 position, the P2 residue is Leu in most cases, and Ser, Ala, or Asn residues are the most prevalent in the P1 position. The other sites show less strict preference, and more different residues occupy these positions. 

### 2.6. Comparison of the Results of NetCorona and 3CLP Predictions 

**Figure 7:**
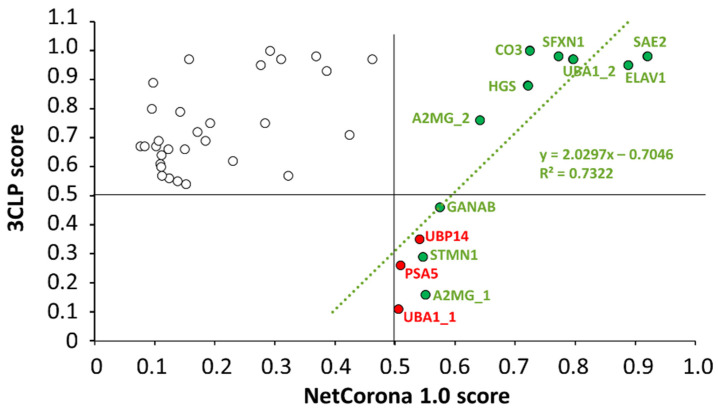
Linear regression analysis of the NetCorona and 3CLP scores. The scores are shown for sequences that were predicted to be cleavage sites either by NetCorona or 3CLP (empty circles). The numerical values are shown in Table 1. The default cutoff of both NetCorona and 3CLP is 0.5, and the >0.5 values (max. score is 1.0) imply possible cleavage at the given positions. The higher the score, the higher the cleavage probability. A regression line (green) was fitted to the data points of cleavage sites for which we observed cleavage in vitro (green circles). Sites that were not cleaved in vitro were omitted from the regression analysis (red circles). Data points of potential cleavage sites predicted by both methods are in the upper right quarter.

The results of the sequence-based in silico analyses were correlated, and a linear regression analysis of the scores obtained by the NetCorona or 3CLP online tools was performed (Figure 7). Only those sites were included in the analysis for which a >0.5 score was calculated either by NetCorona or 3CLP methods, or both. Linear regression analysis revealed no direct correlation between scores if all data points were included (R^2^ = 0.0195), while we observed good correlation (R^2^ = 0.7322) if such sites were analyzed that had >0.5 NetCorona and 3CLP scores and were cleaved by SARS-CoV-2 Mpro in vitro (Figure 3). Interestingly, none of the sites having a <0.6 NetCorona score were predicted as potential cleavage sites by the 3CLP algorithm. In contrast to this, 3CLP predicted high cleavage probability for multiple sites for which a considerably lower NetCorona score (<0.5) was obtained, i.e., for sites that were not identified as potential cleavage sites by NetCorona (Figure 3). For example, in the case of complement C3 (CO3) protein, the 3CLP and NetCorona algorithms identified 8 and 1 potential cleavage sites, respectively (Table 1). The average scores predicted for the seven different sites were 0.749 (3CLP) and 0.174 (NetCorona), indicating that the different methods may identify the same SARS-CoV-2 Mpro sites with considerably different likelihood. There were six cleavage sites that had >0.5 NetCorona and <0.5 3CLP scores (Figure 7). Three of these sites were proved to be cleaved by SARS-CoV-2 Mpro, while the other three were not processed in vitro. This implies that sites having NetCorona scores close to the 0.5 thresholds (between 0.50–0.55) can be identified with relatively lower reliability (e.g., PSA5, UBP14, and UBA1_1).

For those cleavage sites that were processed most efficiently by SARS-CoV-2 Mpro in vitro (ELAV1, HGS, SFXN1, UBA1_2) (Figure 3), both NetCorona and 3CLP web servers predicted high scores (Figure 7), indicating that the most efficient sites can be identified reliably by both methods. Although these sites were predicted to have a >0.7 NetCorona score, we found no obligate correlation between the in vitro observed cleavage efficiencies and the in silico predicted cleavage probabilities, as the highest score (0.920) was predicted for the SAE2 cleavage site. However, this substrate showed no such excessive processing (Figure 3).

## 3. Materials and Methods

All materials were obtained from Sigma-Aldrich (St. Louis, MO, USA), otherwise, it is indicated.

### 3.1. Cell Culturing and Transfection

The human embryonic kidney cells HEK293T cells (Invitrogen, Waltham, MA, USA) were cultured in T-75 flasks in 15 mL Dulbecco’s modified Eagle’s medium (DMEM) containing 10% heat-inactivated fetal bovine serum (FBS; Gibco, Paisley, UK), 1% L-glutamine and 1% penicillin–streptomycin; in a humidified incubator with 5% CO_2_ at 37 °C. 

A day before transfection, HEK293T cells were transferred into a new T-75 flask to achieve a 70–80% confluence on the next day. The pcDNA3.1(+) mammalian expression plasmid coding for the non-tagged SARS-CoV-2 Mpro (GenBank: MT291835.2) was an in-house stock [32]. An empty pcDNA3.1(−) plasmid containing no insert was used as a mock control. HEK293T cells were transfected at 70% confluency with 10 μg plasmid using poly-ethyleneimine (PEI) based on the protocol described previously [32]. The PEI solution containing 150 mM NaCl was added to the plasmids, followed by incubation at room temperature for 20 min. After removing the medium, the cells were washed once with phosphate-buffered saline (PBS), then 5 mL FBS-free DMEM medium was added to the cells. The transfection solution (plasmid DNA+PEI) was added to the medium dropwise; after this, the cells were incubated at 37 °C for 5 h. The transfection mixture was removed carefully, and the medium was replaced with 15 mL DMEM containing 10% FBS, 1% penicillin-streptomycin, and 1% L-glutamine, followed by incubation at 37 °C for a total of 24 h. After incubation, the cell culture medium was removed, and the cells were washed with PBS and trypsinized. After centrifugation (Eppendorf centrifuge 5810R, Germany, 160× *g*, 5 min, room temperature), the pellets were re-suspended, and the cells were counted using 0.4% Trypan Blue stain. The cells were washed three times with ice-cold PBS, then centrifuged at 160× *g*, 4 °C for 10 min. The cell pellet was then stored at −20 °C until use.

### 3.2. Sample Preparation and Protein Labelling 

Mock- and SARS-CoV-2 Mpro-transfected cells were suspended in lysis buffer (30 mM Tris, 7 M urea, 2 M thiourea, 4% *w*/*v* CHAPS, pH 8.5) containing complete EDTA-free protease inhibitor cocktail (Roche). After vortexing, the samples were sonicated (3 × 30 s), followed by centrifugation (12,000× *g*, 4 °C, 5 min). The supernatants were purified using ReadyPrep 2-D CleanUp Kit (Bio-Rad, #163-2130, Hercules, CA, USA) according to the manufacturer’s instructions. After repeated centrifugation, the dried pellet was re-suspended in 400 μL rehydration buffer (7 M urea, 2 M thiourea, 4% (m/v) CHAPS, 1% DTT, 2 *v/v*% Bio-Lyte), followed by staining the samples with three different cyanine dyes (Lumiprobe; Hannover, Germany) according to the manufacturer’s instructions. Then, 1 mM stock solutions of Cyanine2 (Cy2), Cyanine3 (Cy3), and Cyanine5 (Cy5) dyes (Lumiprobe, #1A041, #1B041, and #1C041, respectively) were prepared by adding 1 µL dimethylformamide (DMF) per 1 nmol of each dye. The Cy-dye working solutions were prepared by diluting the stock solutions with DMF to 0.4 mM. The pH of protein samples to be analyzed was set to 8.5 using 1.5 M Tris buffer, then 2.7 µL Cy-dye working solution was added, and samples were incubated for 30 min in the dark at room temperature. Cy3 was used for the mock, Cy5 for SARS-CoV-2 Mpro-transfected cells, and Cy2 for the pooled internal control generated by mixing the mock and Mpro-transfected samples in 1:1 ratio. The reaction was stopped by the addition of 2.7 µL 10 mM lysine (Molar Chemicals; Halásztelek, Hungary), and the Cy2, Cy3, and Cy5-labeled samples were pooled to have 400 µg final total protein content. 

### 3.3. Two-Dimensional Difference Gel Electrophoresis (2D-DIGE)

The mixture of differentially labeled samples was subjected to 2DE using the method described previously [33]. Samples were loaded onto 24 cm-long, pH 3–10 IPG strips with immobilized pH gradient (Bio-Rad, Hercules, CA, USA). Three biological replicates were used and run together on the same day. The gels were scanned on Pharos FX Plus Molecular Imager (Bio-Rad, Hercules, CA, USA) using Quantity One 4.6.7 software (Bio-Rad, Hercules, CA, USA). For the detection of fluorescent signals, 488/530 nm, 532/605 nm, and 635/695 nm excitation/emission wavelengths were used for the Cy2, Cy3, and Cy5 dyes, respectively. After scanning, the gels were stained with in-house prepared ruthenium(II)-tris-bathophenanthroline-disulphonate (RuBPS) fluorescent dye [33,34] and scanned at 532/615 nm wavelength. The scanning resolution was 100 µm in all cases. 

### 3.4. Quantitative Analysis and Statistics

Gels images were analyzed with Delta2D 4.8.2 software (Decodon, Germany) as described earlier [33]. We applied an in-gel standard warping strategy and exact mode matching protocol and prepared fused images using the individual Cy-scanned gels. The fold change of the mock and Mpro-transfected samples was calculated, and the significance of differences was assessed automatically by the Delta2D software using Student’s *t*-test.

### 3.5. In-Gel Digestion of Proteins

Protein spots showing significant differences between the studied groups were excised from the gel and subjected to in-gel trypsin digestion. The spots were destained using a 1:1 ratio of 25 mM ammonium bicarbonate (pH 8.5) and 50% acetonitrile followed by digestion with 100 ng stabilized MS grade trypsin (ABSciex, Framingham, MA, USA) at 37 °C overnight. The reaction was stopped by the addition of concentrated formic acid. The tryptic peptides were extracted from the gel pieces, dried in a vacuum concentrator (Thermo Scientific, Waltham, MA, USA), and kept at −20 °C until mass spectrometric (MS) analysis.

### 3.6. Liquid Chromatography-Mass Spectrometry Analysis

For protein identification by liquid chromatography with tandem MS (LC-MS/MS), the peptides were re-dissolved in 10 μL 1% formic acid (VWR Ltd., Radnor, PA, USA). They were separated in a 180 min water/acetonitrile gradient using an Easy nLC 1200 nano UPLC (Thermo Scientific, Waltham, MA, USA). The peptide mixtures were desalted in an ACQUITY UPLC Symmetry C18 trap column (20 mm × 180 µm, 5 μm particle size, 100 Å pore size; Waters, Milford, MA, USA), followed by separation in a nanoACQUITY Peptide BEH C18 analytical column (150 mm × 75 μm, 1.7 μm particle size, 130 Å pore size; Waters, Milford, MA, USA). The chromatographic separation was performed using a gradient of 5–7% solvent B over 5 min, followed by a rise to 15% of solvent B over 50 min, and then to 35% solvent B over 60 min. Thereafter, solvent B was increased to 40% over 28 min and then to 85% over 5 min, followed by a 10 min rise to 85% of solvent B, after which the system returned to 5% solvent B in 1 min for a 16 min hold-on. Solvent A was 0.1% formic acid in LC water (Sigma, St. Louis, MO, USA); solvent B was 95% acetonitrile (Sigma, St. Louis, MO, USA) containing 0.1% formic acid. The flow rate was set to 300 nL/min.

Data-dependent acquisition experiments were carried out on an Orbitrap Fusion mass spectrometer (Thermo Scientific, Waltham, MA, USA). The 14 most abundant multiply charged positive ions were selected from each survey MS scan, using a scan range of 350–1600 *m/z* for MS/MS analyses (Orbitrap analyzer resolution: 60,000, AGC target: 4.0 × 10^5^, acquired in profile mode). Collision-induced dissociation (CID) fragmentation was performed in the linear ion trap with 35% normalized collision energy (AGC target: 2.0 × 10^3^, acquired in centroid mode). Dynamic exclusion was enabled during the cycles (exclusion time: 45 s).

### 3.7. Protein Identification

The acquired LC-MS/MS data were used for protein identification with the help of MaxQuant 1.6.2.10 software [35] searching against the Human SwissProt database (release: April 2021, 20,376 sequence entries) and the contaminants database provided by the MaxQuant software. Cys carbamidomethylation, Met oxidation, and N-terminal acetylation were set as variable modifications. A maximum of two missed cleavage sites were allowed. Results were imported into Scaffold 5.0.1 software (ProteomeSoftware Inc., Portland, OR, USA). Proteins were accepted with at least 3 identified peptides using a 1% protein false discovery rate (FDR) and 95% peptide probability thresholds.

### 3.8. Western-Blot

Western blot was applied to detect SARS-CoV-2 Mpro in the lysate of transfected HEK293T cells 24 h post-transfection. The cell lysates were supplemented with 6X SDS loading dye and incubated at 95 °C for 10 min. After SDS-PAGE using 12% polyacrylamide gel, the proteins were transferred onto a nitrocellulose membrane at 100 V for 1 h. The membrane was then blocked using 3% powder of non-fat dry milk in Tris-buffered saline (TBS) complemented with Tween20 (TTBS), pH 7.5) for 1 h at room temperature, followed by incubation with rabbit anti-SARS-CoV-2 Mpro polyclonal antibody (Thermo Fischer, PA5-116940) at 4 °C overnight. The primary antibody was applied in a 1:500 dilution, and TTBS containing 0.1 m/v% powder of non-fat dry milk was used for dilution. After washing the membrane three times with TTBS for 15 min, it was incubated with goat anti-rabbit IgG (HRP-conjugate; Bio-Rad #1706515) secondary antibody (1:5000 dilution in TTBS containing 0.1% dry milk) for 1 h at room temperature. The membrane was subsequently washed three times with TTBS, followed by detecting the proteins on the membrane by SuperSignal West Pico chemiluminescent substrate (Thermo Fisher, 34580) using Azure 600 imaging system (Azure Biosystems, Dublin, CA, USA).

### 3.9. Structural Coordinates and Cleavage Site Prediction

The coordinate files were obtained from the RCSB Protein Data Bank [36] and AlphaFold Protein Structure Database (AlphaFold DB, https://alphafold.ebi.ac.uk) (accessed on 8 February 2022) [25]. Molecular visualizations were made by PyMol Molecular Graphics System (Version 1.3 Schrödinger, LLC, New York, NY, USA). The protein sequences were downloaded from the UniProt database [37] (date of last accession: 30 June 2022). The SARS-CoV-2 Mpro cleavage sites and cleavage probabilities were predicted using NetCorona v. 1.0 [13] and the 3CLP (an online tool for predicting coronavirus 3CL protease cleavage sites) [14] web servers. The NetCorona web server is available at https://services.healthtech.dtu.dk/service.php?NetCorona-1.0, while the 3CLP at http://www.computationalbiology.cn/3CLPHost/home.html (accessed on 10 July 2022).

### 3.10. Preparation of Recombinant Protein Substrates

The expression plasmids of His_6_-MBP-mEYFP protein substrates were prepared based on the methods described previously [12]. The oligonucleotide primers coding the cleavage site sequences are shown in Table S2. The success of cloning was confirmed by a DNA sequencing service (Eurofins Genomics Germany GmbH; Ebersberg, Germany). The pDest-His_6_-MBP-mEYFP expression plasmids coding for the cleavage site sequences were transformed into BL21(DE3) *E. coli* cells, and protein expression and purification were performed using the protocols described previously [29,30,38]. The buffer environment of the recombinant His_6_-MBP-mEYFP substrates was changed to distilled water using 10K Amicon tubes (Merck-Millipore, Burlington, MA, USA). The purified proteins were used as substrates of SARS-CoV-2 Mpro in cleavage reactions. The expression plasmid coding for the His_6_-MBP-TSAVLQ*SGFRKM-mEYFP substrate was prepared as described previously [12].

### 3.11. Cleavage Reactions 

The SARS-CoV-2 Mpro was expressed and purified as described previously, and we used the previously optimized conditions for cleavage reactions [12]. Briefly, the reaction mixture contained the purified SARS-CoV-2 Mpro (dialyzed against 20 mM Tris, 150 mM NaCl, 1 mM EDTA, 1 mM DTT, pH 7.8 buffer), a recombinant His_6_-MBP-mEYFP substrate (in distilled water), and the cleavage buffer (20 mM Tris, 100 mM NaCl, pH 7.8). The reaction mixtures were incubated at 37 °C for 1 h. The uncleaved substrates and cleavage products were separated by SDS-PAGE using 14% polyacrylamide gel, using denaturing conditions for sample preparation. For protein detection, the recombinant proteins were renatured in the gels by rinsing the gels in distilled water, followed by visualization of the fluorescent proteins in the unstained gel using UV transillumination [29]. In addition, the gels were stained with Coomassie dye as well. AlphaImager gel documentation system (ProteinSimple) was used for gel imaging.

### 3.12. Cleavage Site Identification by MALDI-TOF MS

Cleavage positions within the predicted target sequence were identified by analysis of the substrate controls and reaction mixtures (incubated for 4 h) by matrix-assisted laser desorption/ionization time-of-flight mass spectrometry (MALDI-TOF MS). The samples were concentrated and desalted by a C4 ZipTip pipette tip (Sigma-Aldrich, St. Louis, MO, USA) according to the manufacturer’s instructions. The matrix was 2,5-dihydroxybenzoic acid (DHB) (100 mg/mL) dissolved in 50% aqueous acetonitrile with 0.1% TFA. 1.0 µL sample was mixed with 0.5 µL matrix solution on a plate and was allowed to air-dry.

The MALDI-TOF MS analyses were carried out on an Autoflex Speed MALDI-TOF mass spectrometer (Bruker Daltonik, Bremen, Germany) equipped with a solid phase laser (355 nm). The linear mode was applied with the voltages of 19.5 kV and 18.3 kV for ion source 1 and ion source 2, respectively. The flexAnalysis software evaluated the spectra (Bruker Daltonik, Bremen, Germany).

## 4. Discussion

We aimed to identify proteins in HEK293T human kidney cells, which are substrates of SARS-CoV-2 Mpro. For this purpose, the cells were transfected with a plasmid coding for the Mpro, and mock-transfected cells were used as control. Changes in the cellular proteomes were determined by comparing the protein contents of the mock-transfected and Mpro-expressing cells using 2D-DIGE. After MS-based identification of the proteins from the spots that showed significantly different intensities, the protein sequences were subjected to in silico cleavage site prediction. Based on the results of the predictions, recombinant substrates containing the putative cleavage sites were designed, and cleavages of the candidate target sites were then tested in vitro, followed by the determination of cleavage positions within the predicted sequences.

It is known that COVID-19 is a multiorgan disease, the incidence of acute kidney injury has also been reported for SARS-CoV-2 infected patients [39], and human kidney tubules were found to be directly infected by SARS-CoV-2 [40]. In accordance with this, HEK293T cells may be efficiently used contextually to study the effects of viral infection, including that of the Mpro. In addition, the HEK293T cell line was chosen for the investigation of Mpro’s substrates since this cell line has already been successfully used to identify cellular targets of SARS-CoV-2 Mpro [20,41]. Changes in the cellular proteomes were investigated 24 h post-transfection. This incubation time was found to enable the expression of Mpro in HEK293T cells at a sufficient level, in agreement with our previous cell culture-based studies [32]. In addition, the same cell line and incubation time were also applied for the proteomic identification of Mpro’s host substrates [41]. This relatively short incubation time was considered to represent proteolytic events that may occur in the early phase of the infection. In this work, we successfully identified host targets of Mpro, which have been identified previously by other studies, indicating that the applied cell culturing and transfection conditions were suitable.

For the in silico prediction of cleavage sites, we applied the NetCorona 1.0 and 3CLP web servers. The results obtained by the different algorithms for all the putative cleavage sites (for which both methods predicted a value higher than the 0.5 thresholds) showed no correlation. In contrast, the prediction results were in agreement for sites that were cleaved by the SARS-CoV-2 Mpro in vitro (Figure 7). The 3CLP online tool overestimated the number of potential cleavage sites compared to the NetCorona 1.0 web server (37 and 13 sites, respectively) (Table 1). Some of the cleavage sites that were found to be cleaved by the SARS-CoV-2 Mpro in vitro were not identified by 3CLP, and sequences having a <0.6 NetCorona score were also not predicted to be cleavage sites by the 3CLP algorithm. The prediction potentials of the NetCorona and 3CLP online tools cannot be compared reliably based on our results, as we performed predictions and cleavage reactions only for a limited set of candidate cleavage sites. Consequently, a more detailed experimental study may better reveal the 3CLP’s prediction potential. To our knowledge, our study is the first to prove the in vitro processing of cleavage sites that were predicted by the 3CLP online tool.

The NetCorona prediction approach was primarily designed for the prediction of SARS-CoV cleavage sites. Nevertheless, multiple studies utilized its prediction potential to analyze those of SARS-CoV-2 Mpro [12,41,42,43]. It is important to note that a limitation of the currently available versions of NetCorona and 3CLP online tools is that they can only identify cleavage sites that contain Gln residue in the P1 position [13,14]. Although histidine may also occupy this position in the minorities of SARS-CoV Mpro [44,45,46] and SARS-CoV-2 Mpro cleavage sites [19,20], no such prediction algorithms have been designed for the reliable identification of P1 His-containing cleavage sites so far. Regarding estimating the prediction potentials for NetCorona and 3CLP online tools in the context of SARS-CoV-2, it is important to note that none of these methods is specific for its Mpro. Based on the known cleavage site sequences, the already existing methods may be improved, and new methods may also be developed. Therefore, the cleavage sites we describe in this paper may aid the design of more accurate and reliable prediction algorithms.

Besides identifying previously known cleavage sites, we provided experimental evidence for the cleavages of previously unknown cleavage sites. Although the predicted target cleavage sites—incorporated into the multidomain protein substrates—may be processed by SARS-CoV-2 Mpro in vitro, not all cleavages may necessarily occur in the properly folded full-length proteins in vivo. It needs to be considered while estimating cleavage probabilities based on crystallographic or modeled structural coordinates that due to the proteins’ structural flexibility and domain movements, the respective arrangement of secondary structural elements and the accessibility of the predicted sites may be potentially different in solution and the rigid molecule crystals. 

In agreement with the results of studies that aimed to identify SARS-CoV-2 proteolytic substrates [9,12,42], our results confirm that it is important to determine the structural characteristics of the candidate cleavage sites that were predicted based on the protein sequences. Scott et al. have used a highly similar in silico methodology for predicting SARS-CoV-2 Mpro cleavage sites, which included comparing viral and human protein sequences, cleavage site prediction by NetCorona webserver, and consideration of structural characteristics of the potential target sites [43]. They predicted that the CTBP1, CTBP2, UBA1, ELAV1, PSA5, CO3, SAE2, A2MG, and UBP14 proteins contain at least one cleavage site of SARS-CoV-2 Mpro. In agreement with this, the existence of the SARS-CoV-2 Mpro cleavage site in CTBP proteins was predicted by our former SSHHPS analysis, and our study provided experimental evidence for the cleavage of the predicted cleavage site sequence [12]. The UBA1 and ELAV1 proteins were also predicted by Scott et al. to contain potential substrates of SARS-CoV-2 Mpro (UBA1_2 cleavage site in UBA1 protein) [43]. In this work, we confirmed that the enzyme could efficiently cleave the sites within these proteins. The GANAB, STMN1, ELAV1, SAE2, and UBA1 proteins were previously identified as host substrates of SARS-CoV-2 Mpro in the pulmonary cell line cells [19]. Here, we proved the cleavages at the predicted sites in vitro, in the case of STMN1, ELAV1, and UBA1 proteins, while new cleavage sites were also identified in A2MG, CO3, GANAB, and SAE2 proteins. The agreement of these data proves a good prediction potential of the NetCorona algorithm and the applicability of the recombinant substrate-based experimental approach for cleavage site identification. In addition, identifying non-cleaved (wild-type or modified) sequences capable of inhibiting the Mpro may provide valuable information for inhibitor design.

The candidate substrates of SARS-CoV-2 Mpro we identified have diverse biological functions based on the experimentally determined protein–protein interactions available in the STRING database [47]. Most of the target proteins (A2MG, CO3, ELAV1, GANAB, HGS, and UBA1) can be categorized into the extracellular region and cysts based on the cellular compartment categories of the Gene Ontology database [48]. The UBA1 and HGS proteins directly interact with each other, and both of them indirectly interact with SAE2.

Small ubiquitin-like modifier (SUMO) proteins mediate post-translational modification of cellular proteins involved in various downstream cellular activities [49,50]. Naturally, dysregulation, or hijacking, of the SUMOylation process is an attractive target for many viruses to ensure effective infection and replication in the host cell. Many viruses were found to manipulate SUMOylation in order to modulate host antiviral responses and enhance pathogenesis [51], and SARS-CoV-2 is no exception. The non-structural protein 5 (nsp-5) of SARS-CoV-2 was found to increase the stability and protein level of the mitochondrial antiviral-signaling protein (MAVS) through modulation of its SUMOylation, resulting in the increased activation of NF-κB signaling pathway [52]. SUMO-activating enzyme subunit 2 (SAE2) forms a heterodimer with SUMO-activating enzyme subunit 1 (SAE1), functioning as an E1-activating enzyme that results in the activation of mature SUMO proteins in an ATP-dependent manner during the process of SUMOylation [50]. Therefore, disruption to the formation of the SUMO E1 heterodimer may result in the downstream accumulation of unmodified SUMO substrates, enhancing viral pathogenicity, at least in the case of adenoviruses, although many viruses are also likely to be utilizing this mechanism [53]. Here, our experiments indicated that SAE2 might harbor a cleavage site for SARS-CoV-2. However, whether or not the processing of SAE2 in the context of infection results in decreased quantity of the protein; or disruption to the heterodimer formation remains to be elucidated.

Complement system hyperactivation is now regarded as a hallmark of COVID-19. Proteolytic processing of complement CO3 protein results in the production of an array of cleavage products essential to activating the classical pathways of complement activity [54]. In fact, excessive activation and consumption of CO3 were found to correlate with disease severity and mortality in patients hospitalized with COVID-19 [55,56]. While the S and nucleocapsid proteins of SARS-CoV-2 were found to directly mediate activation of the complement pathways through the lectin pathway components or dysregulation of the alternative pathways [57,58], so far, according to our knowledge, Mpro has not been implicated in its activation. The fact that CO3 was found to contain a potential cleavage site for Mpro is indeed intriguing. It only raises more questions about the possibility that Mpro might contribute to the activation of the complement pathway. At least, the identification of a new cleavage site representing a sequence motif of CO3 can help better understand Mpro’s specificity.

The 2D-DIGE analysis revealed changes in the levels of proteins that were predicted to contain no cleavage sites of Mpro (Table 1), or their candidate cleavage site sequence were not cleaved in vitro (Figure 3). The observed changes in the protein levels were considered to be caused by the expression of SARS-CoV-2 Mpro rather than by the transfection itself since an empty pcDNA3.1 vector transfected the control cells. Although expression of the recombinant Mpro may have affected the levels of multiple cellular proteins, we assumed that changes in the cellular proteome could not be interpreted exclusively at the level of Mpro expression since the changes induced by the virions are more complex due to the predisposition of the infected cells to the complete set of viral proteins [59,60].

A possible limitation of our study may be the relatively lower sensitivity of the 2D-DIGE compared to the N-terminomics [19,20,61]. Thus, the number of the Mpro substrates we identified in this study was lower. However, we investigated the proteolysis of intact (i.e., non-denatured or pre-digested) proteins in the cellular environment rather than digesting cell lysates. Therefore, our experimental approach was considered to reveal physiologically relevant cleavage events, at least in the context of HEK293T cells. Moreover, investigation of additional pharmacologically relevant cell lines (such as pulmonary, neuronal, and cardiovascular) may also be desired to better map the substrate profile of the viral protease. The methodology we applied was found to be useful for the prediction of SARS-CoV-2 Mpro cleavage sites. The identification of cellular targets and cleavage site sequences may aid a better understanding of viral pathogenesis and enzyme specificity, respectively.

## Figures and Tables

**Table 1:** Predicted cleavage sites of SARS-CoV-2 Mpro in cleavage sites in potential substrates identified by 2D-DIGE. Proteins identified in the spots with statistically significant differences between mock- and SARS-CoV-2 Mpro-transfected cells are listed. The name of the proteins and the unique identifiers are shown based on the UniProt database. The cleavage sites were predicted using NetCorona and 3CLP web servers. Potential cleavage sites which were identified by both methods are highlighted with a light green background, while the light orange background indicates that none of the algorithms predicted the existence of any cleavage site. Sequences are shown only in those cases if they were predicted to contain a potential cleavage site. White background indicates that the given site was identified by only one of the algorithms. ^#^ These cleavage sites have been identified previously by Koudelka et al. [19].

	Protein		NetCorona Prediction Output			3CLP Prediction Output		
Spot	(UniProt ID; Name)	Cleavage Position	Cleavage	Score	Cleavage Site	Cleavage	Score	Cleavage Site
3, 4	P11021; BIP	Q401	No	0.276	-	Yes	0.95	AAVQ*AG
9, 10	P09651; ROA1	-	No	-	-	No	-	-
2	P55072; TERA	Q421	No	0.368	-	Yes	0.98	AALQ*AI
11	P18669; PGAM1	-	No	-	-	No	-	-
1	Q14697; GANAB	Q92	No	0.142	-	Yes	0.79	LELQ*GL
		Q343 ^#^	No	0.143	-	No	0.20	-
		Q780	No	0.122	-	Yes	0.66	YDIQ*SY
		Q906	Yes	0.574	AVVLQ*TKGSP	No	0.46	-
15	P16949; STMN1	Q18 ^#^	Yes	0.546	RASGQ*AFELI	No	0.29	-
9, 10	Q9H9B4; SFXN1	Q303	Yes	0.772	EAELQ*AKIQE	Yes	0.98	AELQ*AK
9, 10	Q15717; ELAV1	Q87 ^#^	Yes	0.888	GLRLQ*SKTIK	Yes	0.95	LRLQ*SK
9, 10	Q99623; PHB2	-	No	-	-	No	-	-
1	O95757; HS74L	Q470	No	0.150	-	Yes	0.66	VFPQ*SD
		Q585	No	0.102	-	Yes	0.67	LPIQ*SS
12	P28066; PSA5	Q114	Yes	0.509	ESVTQ*AVSNL	No	0.26	-
		Q164	No	0.184	-	Yes	0.69	TFVQ*CD
3	P01024; CO3	Q109	No	0.292	-	Yes	1.00	VTVQ*AT
		Q126	Yes	0.724	LVSLQ*SGYLF	Yes	1.00	VSLQ*SG
		Q392	No	0.157	-	Yes	0.97	VAVQ*GE
		Q398	No	0.283	-	Yes	0.75	DTVQ*SL
		Q661	No	0.171	-	Yes	0.72	AELQ*CP
		Q1277	No	0.124	-	Yes	0.56	MVFQ*AL
		Q1299	No	0.112	-	Yes	0.57	VSLQ*LP
		Q1553	No	0.076	-	Yes	0.67	VKVQ*LS
1	P19338; NUCL	Q558	No	0.095	-	Yes	0.80	LELQ*GP
7	P05455; LA	Q358	No	0.462	-	Yes	0.97	VQFQ*GK
5	P54578; UBP14	Q40	No	0.083	-	Yes	0.67	TGVQ*PA
		Q121	No	0.106	-	Yes	0.69	ATVQ*CI
		Q306	Yes	0.541	SPTLQ*RNALY	No	0.35	-
		Q421	No	0.424		Yes	0.71	YDLQ*AV
3	P13667; PDIA4	Q377	No	0.111	-	Yes	0.64	MDVQ*GS
3	Q9NY33; DPP3	-	No	-	-	No	-	-
1	P36776; LONM	-	No	-	-	No	-	-
14	P30048; PRDX3	-	No	-	-	No	-	-
3	P00738; HPT	-	No	-	-	No	-	-
3	P01023; A2MG	Q592	No	0.230	-	Yes	0.62	AAPQ*SV
		Q602	Yes	0.550	RAVDQ*SVLLM	No	0.16	-
		Q827	No	0.152	-	Yes	0.54	VSVQ*LE
		Q960	Yes	0.641	GSAMQ*NTQNL	Yes	0.76	SAMQ*NT
		Q1281	No	0.386	-	Yes	0.93	VTIQ*SS
13	P15374; UCHL3	-	No	-	-	No	-	-
2	Q9UBT2; SAE2	Q82 ^#^	No	0.302	-	No	0.28	-
		Q288	No	0.310	-	Yes	0.97	AEVQ*SQ
		Q518	Yes	0.920	GSRLQ*ADDFL	Yes	0.98	SRLQ*AD
1	O14964; HGS	Q444	No	0.192	-	Yes	0.75	SLFQ*SI
		Q571	Yes	0.721	YAQLQ*AMPAA	Yes	0.88	AQLQ*AM
		Q649	No	0.109	-	Yes	0.61	AAPQ*AQ
3	P10909; CLUS	-	No	-	-	No	-	-
1, 7	P22314; UBA1	Q254	No	0.097	-	Yes	0.89	SEVQ*GM
		Q334	No	0.138	-	Yes	0.55	IGFQ*AL
		Q463	Yes	0.506	GSDLQ*EKLGK	No	0.11	-
		Q792 ^#^	Yes	0.796	ATFLQ*SVQVP	Yes	0.97	TFLQ*SV
		Q815	No	0.322	-	Yes	0.57	QELQ*SA
		Q961	No	0.110	-	Yes	0.60	FEVQ*GL

**Table 2:** Summary tables of predicted SARS-CoV-2 Mpro cleavage sites. * The substrates and cleavage sites—studied in this work—were compared to those identified previously by Koudelka et al. [19]. P1 Gln residues are bold. ** To prove that the candidate sites are susceptible to proteolysis, cleavage reactions were performed using His_6_-MBP-mEYFP recombinant proteins as substrates of SARS-CoV-2 Mpro. Representative cleavage reactions are shown in Figure 3. If structural coordinate was not available in Protein Data Bank, the structure predicted by AlphaFold was used (AF).

Spot Number	Protein (UniProt ID and Name)	Cleavage Site		Calculated Mw (kDa)		NetCorona	In Vitro Cleavage **	Coordinate File	
		Sequence	Identified as Substrate/Cleavage Site *	Full- Length Protein	Cleavage Products	Cleavage Probability		PDB ID	Ref.
3	P01023; A2MG	RAVDQ*SVLLM	No/No	163.3	67.0/96.3	+	Yes	4ACQ	[22]
		GSAMQ*NTQNL	No/No		106.1/57.2	++	Yes		
	P01024; CO3	LVSLQ*SGYLF	No/No	187.2	13.6/173.6	+++	Yes	5FOA	[23]
9, 10	Q15717; ELAV1	GLRLQ*SKTIK	Yes/Yes	36.1	9.7/26.4	++++	Yes	4ED5	[24]
1	Q14697; GANAB	AVVLQ*TKGSP	Yes/No	106.9	102.6/4.3	+	Yes	AF	[25]
	O14964; HGS	YAQLQ*AMPAA	No/No	86.2	65.2/21.0	+++	Yes	AF	[25]
12	P28066; PSA5	ESVTQ*AVSNL	No/No	26.4	12.6/13.8	+	No	4R3O	[26]
2	Q9UBT2; SAE2	GSRLQ*ADDFL	Yes/No	71.2	57.7/13.5	+++++	Yes	1Y8Q	[27]
9, 10	Q9H9B4; SFXN1	EAELQ*AKIQE	No/No	35.6	33.3/2.3	+++	Yes	AF	[25]
15	P16949; STMN1	RASGQ*AFELI	No/Yes	17.3	2.0/15.3	+	Yes	AF	[25]
1, 7	P22314; UBA1	GSDLQ*EKLGK	No/No	117.9	-	+	No	6DC6	[28]
		ATFLQ*SVQVP	Yes/Yes		87.6/30.3	+++	Yes		
5	P54578; UBP14	SPTLQ*RNALY	No/No	56.1	-	+	No	2AYN	[11]

## Data Availability

Additional data are available from the authors upon request.

## Supplementary Materials

The following supporting information can be downloaded at: https://www.mdpi.com/article/10.3390/ijms24043236/s1.
