# Supplementary material for: Identification of SARS-CoV-2 Main Protease (Mpro) Cleavage Sites Using Two-Dimensional Electrophoresis and In Silico Cleavage Site Prediction
Source: Int J Mol Sci. 2023 Feb 6;24(4):3236. doi: 10.3390/ijms24043236 (PMC9965337; doi:10.3390/ijms24043236)
Supplement: Supplementary file 1 [file ijms-24-03236-s001.zip › Table S1.pdf]

Table S1. The calculated and measured m/z values of the recombinant substrates and their cleavage products. The m/z values were determined experimentally by MALDI-TOF MS measurements. Asterisk denotes that only doubly charged species were detected, with low intensity. In the case of complete substrate turnover, the full-length substrates were not detectable (n.d.) in the samples. If a substrate was not cleaved, cleavage fragments were not detected.

		A2MG_1	A2MG_2	CO3	ELAV1	GANAB	HGS	PSA5
Calculated masses (Da)	Substrate	72320	72252	72315	72332	72188	72252	72236
	N-terminal fragment	44588	44492	44559	44586	44529	44622	44562
	C-terminal fragment	27751	27777	27774	27764	27677	27648	27691
Measured masses (Da)		**control**	**control**	**control**	**control**	**control**	**control**	**control**
	Substrate	72348	72271	72691	72502	72286	72598	72492*
		**digested**	**digested**	**digested**	**digested**	**digested**	**digested**	**digested**
	Substrate	72333	72425	n.d.	n.d.	72558	n.d.	72471
	N-terminal fragment	44724	44414	44613	44588	44766	44851	n.d.
	C-terminal fragment	27582	27755	27663	27640	27742	27678	n.d.
		**SAE2**	**SFXN1**	**STMN1**	**UBA1_1**	**UBA1_2**	**UBP14**	
Calculated masses (Da)	Substrate	72310	72347	72280	72263	72278	72351	
	N-terminal fragment	44560	44589	44517	44518	44579	44545	
	C-terminal fragment	27768	27776	27780	27762	27717	27825	
Measured masses (Da)		**control**	**control**	**control**	**control**	**control**	**control**	
	Substrate	72379	72442	72302	72195	72286	72664	
		**digested**	**digested**	**digested**	**digested**	**digested**	**digested**	
	Substrate	72270	n.d.	n.d.	72301	n.d.	72567	
	N-terminal fragment	44575	44660	44547	n.d.	44583	n.d.	
	C-terminal fragment	27672	27726	27683	n.d.	27627	n.d.	
