# Supplementary material for: Identification of SARS-CoV-2 Main Protease (Mpro) Cleavage Sites Using Two-Dimensional Electrophoresis and In Silico Cleavage Site Prediction
Source: Int J Mol Sci. 2023 Feb 6;24(4):3236. doi: 10.3390/ijms24043236 (PMC9965337; doi:10.3390/ijms24043236)
Supplement: Supplementary file 1 [file ijms-24-03236-s001.zip › Table S2.pdf]

**Table S2. Oligonucleotide primers coding for the predicted cleavage sites of SARS-CoV-2 Mpro.** After annealing of the complementary oligonucleotide primers, the 5′ and 3′ ends of the resulting short double-stranded DNAs represented the sticky ends complementary to those formed by PacI and NheI restriction endonucleases, respectively. FWD: forward; REV: reverse. # The sequence of the sequencing primer was published previously: Gazda LD, Joóné Matúz K, Nagy T, Mótyán JA, Tőzsér J. Biochemical characterization of Ty1 retrotransposon protease. *PLoS One*. 2020; 15(1): e0227062.

| Protein | Cleavage site | Primer | Oligonucleotide primer sequence |
|---|---|---|---|
| Q14697_GANAB | AVVLQ*TKGSP | FWD | 5′-TAAAGCGGTGGTGCTGCAGACCAAAGGCTCTCCAG-3′ |
| | | REV | 5′-CTAGCTGGAGAGCCTTTGGTCTGCAGCACCACCGCTTTAAT-3′ |
| P16949_STMN1 | RASGQ*AFELI | FWD | 5′-TAAACGTGCGTCTGGCCAGGCGTTTGAACTGATCG-3′ |
| | | REV | 5′-CTAGCGATCAGTTCAAACGCCTGGCCAGACGCACGTTTAAT-3′ |
| Q9H9B4_SFXN1 | EAELQ*AKIQE | FWD | 5′-TAAAGAAGCGGAACTGCAGGCGAAAATCCAGGAAG-3′ |
| | | REV | 5′-CTAGCTTCCTGGATTTTCGCCTGCAGTTCCGCTTCTTTAAT-3′ |
| Q15717_ELAV1 | GLRLQ*SKTIK | FWD | 5′-TAAAGGCCTGCGTCTGCAGTCTAAAACCATCAAAG-3′ |
| | | REV | 5′-CTAGCTTTGATGGTTTTAGACTGCAGACGCAGGCCTTTAAT-3′ |
| P28066_PSA5 | ESVTQ*AVSNL | FWD | 5′-TAAAGAATCTGTGACCCAGGCGGTGTCTAACCTGG-3′ |
| | | REV | 5′-CTAGCCAGGTTAGACACCGCCTGGGTCACAGATTCTTTAAT-3′ |
| P01024_CO3 | LVSLQ*SGYLF | FWD | 5′-TAAACTGGTGTCTCTGCAGTCTGGCTATCTGTTTG-3′ |
| | | REV | 5′-CTAGCAAACAGATAGCCAGACTGCAGAGACACCAGTTTAAT-3′ |
| P54578_UBP14 | SPTLQ*RNALY | FWD | 5′-TAAATCTCCAACCCTGCAGCGTAACGCGCTGTATG-3′ |
| | | REV | 5′-CTAGCATACAGCGCGTTACGCTGCAGGGTTGGAGATTTAAT-3′ |
| P01023_A2MG | RAVDQ*SVLLM | FWD | 5′-TAAACGTGCGGTGGATCAGTCTGTGCTGCTGATGG-3′ |
| | | REV | 5′-CTAGCCATCAGCAGCACAGACTGATCCACCGCACGTTTAAT-3′ |
| | GSAMQ*NTQNL | FWD | 5′-TAAAGGCTCTGCGATGCAGAACACCCAGAACCTGG-3′ |
| | | REV | 5′-CTAGCCAGGTTCTGGGTGTTCTGCATCGCAGAGCCTTTAAT-3′ |
| Q9UBT2_SAE2 | GSRLQ*ADDFL | FWD | 5′-TAAAGGCTCTCGTCTGCAGGCGGATGATTTTCTGG-3′ |
| | | REV | 5′-CTAGCCAGAAAATCATCCGCCTGCAGACGAGAGCCTTTAAT-3′ |
| O14964_HGS | YAQLQ*AMPAA | FWD | 5′-TAAATATGCGCAGCTGCAGGCGATGCCAGCGGCGG-3′ |
| | | REV | 5′-CTAGCCGCCGCTGGCATCGCCTGCAGCTGCGCATATTTAAT-3′ |
| P22314_UBA1 | GSDLQ*EKLGK | FWD | 5′-TAAAGGCTCTGATCTGCAGGAAAAACTGGGCAAAG-3′ |
| | | REV | 5′-CTAGCTTTGCCCAGTTTTTCCTGCAGATCAGAGCCTTTAAT-3′ |
| | ATFLQ*SVQVP | FWD | 5′-TAAAGCGACCTTTCTGCAGTCTGTGCAGGTGCCAG-3′ |
| | | REV | 5′-CTAGCTGGCACCTGCACAGACTGCAGAAAGGTCGCTTTAAT-3′ |
| sequencing primer # | | FWD | 5'-GATGAAGCCCTGAAAGACGCGCAG-3' |
